# Assessment of right atrial dyssynchrony by 2D speckle-tracking in healthy young men following high altitude exposure at 4100 m

**DOI:** 10.1371/journal.pone.0247107

**Published:** 2021-02-18

**Authors:** Chunyan He, Hedong Xiang, Chuan Liu, Shiyong Yu, Jie Yang, Xiaohan Ding, Shizhu Bian, Jihang Zhang, Hu Tan, Jun Jin, Mingdong Hu, Chen Zhang, Rongsheng Rao, Lan Huang

**Affiliations:** 1 Institute of Cardiovascular Diseases of PLA, the Second Affiliated Hospital, Third Military Medical University (Army Medical University), Chongqing, China; 2 Department of Cardiology, the Second Affiliated Hospital, Third Military Medical University (Army Medical University), Chongqing, China; 3 Department of Health Care and Geriatrics, the 940th Hospital of Joint Logistics Support Force of PLA, Lanzhou, China; 4 Department of Respiratory Medicine, the Second Affiliated Hospital, Third Military Medical University (Army Medical University), Chongqing, China; 5 Department of Medical Ultrasonics, the Second Affiliated Hospital, Third Military Medical University (Army Medical University), Chongqing, China; Medical University Innsbruck, AUSTRIA

## Abstract

**Background:**

High altitude exposure induces overload of right-sided heart and may further predispose to supraventricular arrhythmia. It has been reported that atrial mechanical dyssynchrony is associated with atrial arrhythmia. Whether high altitude exposure causes higher right atrial (RA) dyssynchrony is still unknown. The aim of study was to investigate the effect of high altitude exposure on right atrial mechanical synchrony.

**Methods:**

In this study, 98 healthy young men underwent clinical examination and echocardiography at sea level (400 m) and high altitude (4100 m) after an ascent within 7 days. RA dyssynchrony was defined as inhomogeneous timing to peak strain and strain rate using 2D speckle-tracking echocardiography.

**Results:**

Following high altitude exposure, standard deviation of the time to peak strain (SD-TPS) [36.2 (24.5, 48.6) ms vs. 21.7 (12.9, 32.1) ms, p<0.001] and SD-TPS as percentage of R–R’ interval (4.6 ± 2.1% vs. 2.5 ± 1.8%, p<0.001) significantly increased. Additionally, subjects with higher SD-TPS (%) at high altitude presented decreased right ventricular global longitudinal strain and RA active emptying fraction, but increased RA minimal volume index, which were not observed in lower group. Multivariable analysis showed that mean pulmonary arterial pressure and tricuspid E/A were independently associated with SD-TPS (%) at high altitude.

**Conclusion:**

Our data for the first time demonstrated that high altitude exposure causes RA dyssynchrony in healthy young men, which may be secondary to increased pulmonary arterial pressure. In addition, subjects with higher RA dyssynchrony presented worse RA contractile function and right ventricular performance.

## Introduction

High altitude is defined as locations higher than 2500 m above sea level [1], which has been recognized as a physiological challenge to cardiovascular system [2]. Short-term high altitude exposure of lowlanders is characterized by decreased oxygen saturation, sympathetic activation, enhanced ventilation, pulmonary vasoconstriction, and subsequently hypoxic pulmonary hypertension, which increase the work of right-sided heart and may further predispose to arrhythmia at high altitude [1,3–6]. Boos, Holdsworth [4] recorded significant supraventricular arrhythmias in healthy adult men after exposure to over 4100 m above sea level. However, the myocardial mechanical substrate for proarrhythmia at high altitude is unknown. Recent studies have presented a definition of mechanical dyssynchrony as inhomogeneous timing of cardiac mechanical behavior assessed by speckle-tracking echocardiography, which was associated with arrhythmia [7–9]. Thus, it may provide a novel insight to explore the atrial mechanism underlying high altitude-induced arrhythmia.

Cardiac response to high altitude has been regarded as slightly decreased right ventricular (RV) systolic function, even right heart failure, however preserved left ventricular systolic function [10]. Due to the unique arrangement of myofibres, regional myocardial motion and global function are intrinsically linked. Indeed, it has been validated that myocardial mechanical dyssynchrony correlates cardiac mechanics with function as well [11,12]. Our previous study has revealed that high altitude exposure induced RV dyssynchrony, which was related to decreased RV performance [12]. High altitude-induced RV overload directly conducts to right atrium (RA), consequently leading to increased RA pressure. Much like a recent study, Deng, Guo [13] observed pronounced left atrial dyssynchrony in patients with mitral stenosis, which might be through increasing left atrial afterload. Nevertheless, whether high altitude exposure induces RA mechanical dyssynchrony remains unclear.

The atrium plays an indispensable role in modulating cardiac performance as reservoir, conduit and pump [14]. Recent studies demonstrated that atrial dysfunction was sensitive to detect the subclinical abnormality in several diseases [15–21]. With the advent of the speckle-tracking echocardiography technology, we are able to quantitatively assess atrial mechanical function with better reproducibility and less angle-dependence than conventional methods [22]. In this study, we aimed to investigate the characteristics of RA mechanical synchrony after high altitude exposure to 4100 m using 2D speckle-tracking echocardiography and further find its potential determinants.

## Methods

### Study population and procedure

In June 2013, a total of 98 healthy young men, who were permanently living below 500 m, were enrolled the study. The exclusion criteria were any known cardiovascular and pulmonary diseases, previous history of exposure to altitudes above 2500 m above sea level in the past 6 months, and suboptimal quality images. All subjects provided written informed content to participate. The study was performed according to Declaration of Helsinki and received approval by the Clinical Research Ethics Committee of the Third Military Medical University (Army Medical University) (No: 2012015), The experimental protocol was registered under the Chinese Clinical Trial Registration (No: ChiCTR-RCS-12002232, http://www.chictr.org.cn). Authors had no access to information that could identify individual participants during or after data collection. The subjects enrolled our study were transported by bus from Yanggongqiao (Chongqing, China, 400m) to Litang (Sichuan, China, 4100 m) in 7 days.

Clinical examination, transthoracic echocardiography and symptom questionnaire were conducted both at sea level (400 m) and within 5 ± 2 h after arrival at 4100 m. Clinical examination included the measurement of arterial pulse oxygen saturation (Nonin ONYX OR9500, USA). Blood pressure was measured using automatic sphygmomanometer (Omron HEM-6200, Japan) in supine position after resting for 5 minutes. Heart rate (HR) was monitored by the electrocardiogram connected to the ultrasound system during the examination. Acute mountain sickness was defined as Lake Louise score ≥ 3, in the presence of a headache [23]. Besides headache, the symptoms of Lake Louise score included gastrointestinal symptoms, fatigue and dizziness.

### Echocardiographic image acquisition and analysis

Transthoracic echocardiography was performed in the left lateral decubitus position by experienced cardiac sonographer using a commercially available CX50 ultrasound machine (Philips Ultrasound System, Andover, MA, USA) according to the recommendation of the American Society of Echocardiography [24]. The data were saved digitally and analyzed offline using QLAB workstation (version 10.5, Philips Healthcare, Andover, MA, USA).

Ventricular volumes and areas were measured at end-systole and end-diastole to obtain left ventricular ejection fraction and RV fractional area change, respectively [25,26]. RA volumes were automatically calculated by the software using Simpson’s method. RA maximal volume (Vmax) was obtained in ventricular end-systole at the onset of tricuspid valve opening, pre-systolic volume (Vpre) was obtained preceding the P wave and minimal volume (Vmin) was obtained at the onset of tricuspid valve closure [26]. RA volumes were indexed to body surface area. RA total emptying fraction (EFtot) was calculated by (Vmax–Vmin)/Vmax, passive emptying fraction (EFpass) was (Vmax–Vpre)/Vmax, and active emptying fraction (EFact) was (Vpre–Vmin)/Vpre [27].

Peak early diastolic E-wave velocity, peak late diastolic A-wave velocity, peak tricuspid regurgitant velocity and pulmonary artery systolic wave acceleration time were obtained by pulsed-wave Doppler echocardiography. Due to the availability and feasibility of pulmonary artery systolic wave acceleration time in all subjects [28], mean pulmonary arterial pressure (mPAP) was assessed by pulmonary artery systolic wave acceleration time [29]. Systolic pulmonary arterial pressure was assessed using simplified Bernoulli equation: 4×peak tricuspid regurgitant velocity^2^ + 5 mmHg (an estimated central venous pressure) [29].

RV-focused apical four-chamber greyscale images were obtained using 2D speckle-tracking echocardiography with 70–90 frames per second. For evaluating RV longitudinal systolic function, tricuspid annular motion was calculated from average of tricuspid lateral and septal annular displacement using 2D speckle-tracking echocardiography [30], and RV global longitudinal strain (GLS) was assessed automatically in RV six segments by the software [31].

### RA dyssynchrony quantitation

**Fig 1:**
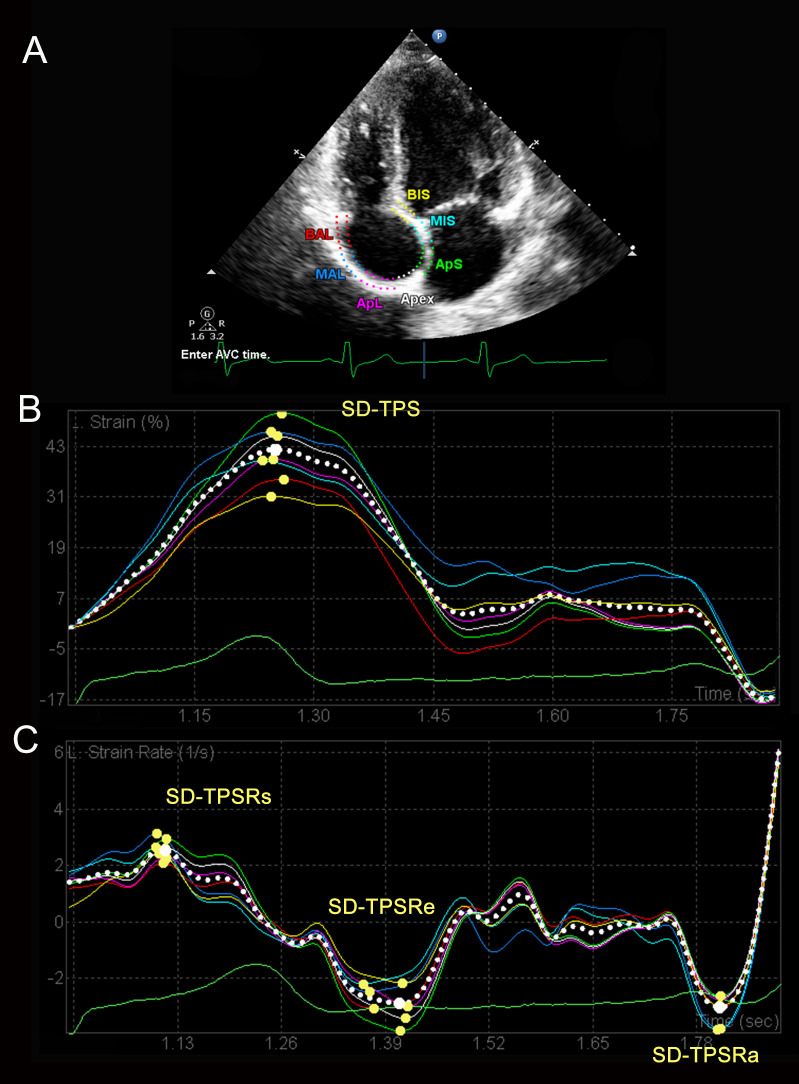
The assessment of right atrial dyssynchrony. (A) Representative 2D speckle-tracking image of RA. RA myocardium is divided into seven segments, and strain (B) and strain rate (C) curves are generated automatically for each segment. RA dyssynchrony is defined as standard deviation of the time to the peak strain or strain rate. RA, right atrium; SD, standard deviation; TPS, time to peak strain; TPSRs, time to peak systolic strain rate; TPSRe, time to peak early diastolic strain rate; TPSRa, time to peak late diastolic strain rate.

RA strain and mechanical dyssynchrony were obtained by 2D speckle-tracking echocardiography. RA endocardial-epicardial borders were automatically traced in four-chamber view and manually adjusted by the operator for optimal quantitation. The frame at QRS wave onset was used as the first reference frame. RA myocardium was automatically divided into seven segments, and strain and strain rate curves were generated for each segment. RA strain during RV systole (represent RA reservoir function) was measured as difference of the strain value at tricuspid valve opening minus RV end-diastole [31]. Based on strain and strain rate curves, the indexes of RA dyssynchrony were assessed as follows (Fig 1) [7].

Standard deviation-time-to-peak strain (SD-TPS, ms): standard deviation of the time to the peak strain in seven segments.

Standard deviation-time-to-peak systolic strain rate (SD-TPSRs, ms): standard deviation of the time to the peak strain rate during RV systole in seven segments.

Standard deviation-time-to-peak early diastolic strain rate (SD-TPSRe, ms): standard deviation of the time to the peak strain rate during RV early diastole in seven segments.

Standard deviation-time-to-peak late diastolic strain rate (SD-TPSRa, ms): standard deviation of the time to the peak strain rate during RV late diastole in seven segments.

Interatrial dyssynchrony was defined as the difference between the time to peak strain rate during ventricular late diastole at left atrial free wall and at RA free wall [32,33].

Higher value was defined as greater degree of mechanical dyssynchrony. We also presented the indexes of dyssynchrony as percentage of R–R’ interval (%).

### Statistical analysis

Continuous variables were expressed as the mean ± standard deviation or median (interquartile range), and categorical variables were expressed as the number with proportions. The comparisons of continuous variables were assessed using the paired t-test or nonparametric test between two groups, and one-way ANOVA with a post hoc test or Kruskal-Wallis test between more than two groups. The comparisons of categorical variables were assessed using chi-square test or Fisher’s exact test. Subjects were graded according to tertiles of RA SD-TPS (%) and interatrial dyssynchrony at high altitude, respectively (grade 1: <33^rd^, grade 2: 33^rd^-66^th^, grade 3: ≥66^th^). Linear trend in continuous variables according to RA SD-TPS (%) grade was tested by the liner regression analysis in Table 2. Univariable linear regression analysis was performed to assess the related variables for RA dyssynchrony, and variables with p<0.1 were entered to the stepwise multivariable linear regression. Statistical analysis was performed using SPSS 22.0 (IBM Corp., Armonk, NY, USA) and Graphpad Prism 7.0 (Inc., La Jolla, USA).

Intra- and inter-observer variabilities for RA function and dyssynchrony were assessed in 10 randomly selected subjects at sea level and high altitude by the same observer and by two independent observers, respectively, using the intra-class correlation coefficient by Cronbach’s α. The value below 0.05 was defined significant for all hypothesis tests.

## Results

### The effect of high altitude exposure on RA mechanical dyssynchrony

**Fig 2:**
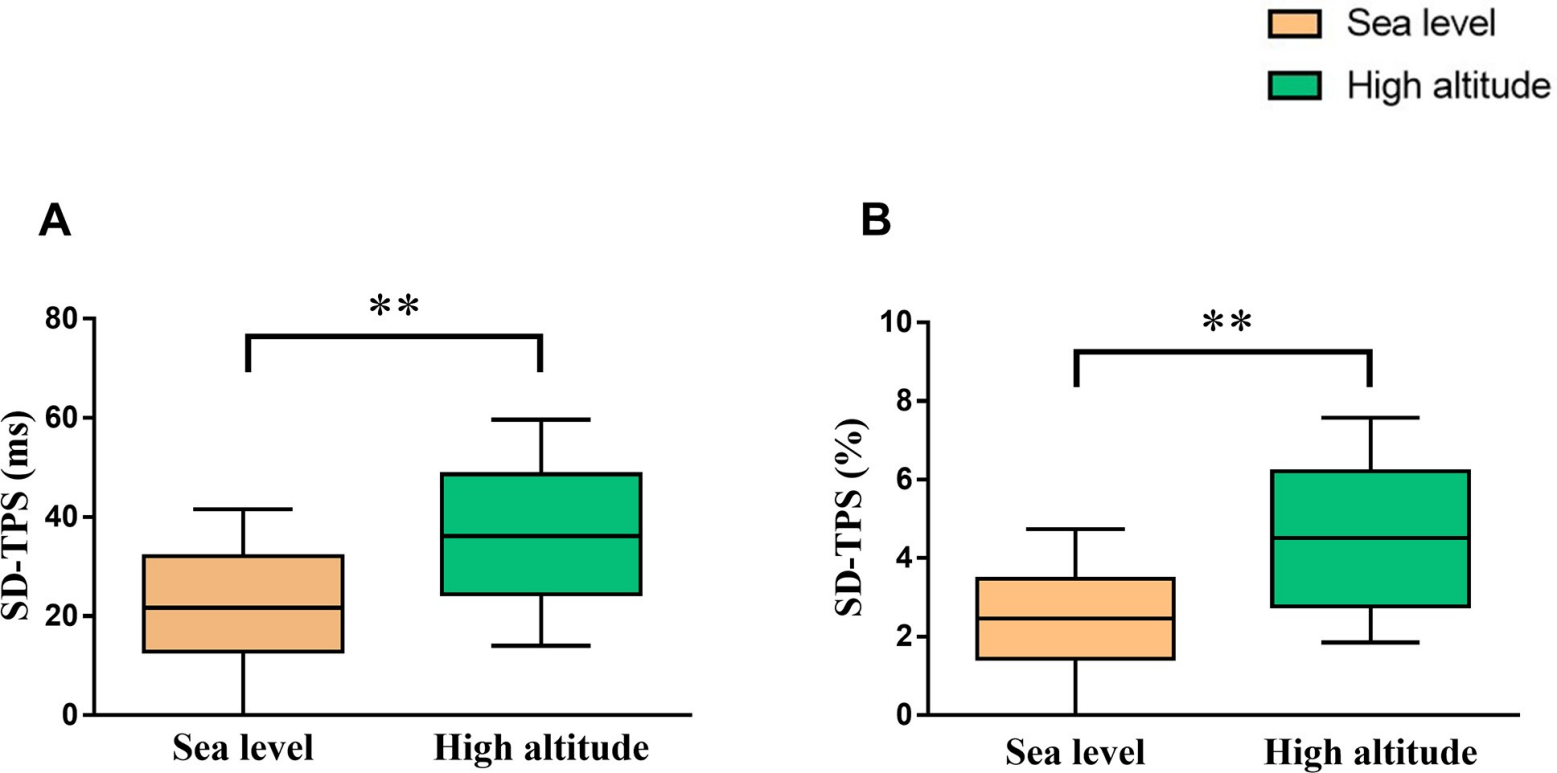
Comparison of right atrial dyssynchrony between at sea level and high altitude. *p<0.05; **p<0.01. Abbreviations as in Fig 1.

A total of 98 young men aged 20.0 (19.0, 22.0) years with body mass index of 21.1 (19.7, 22.4) kg/m^2^ were enrolled the study. The quality of images was sufficient to analyze RA mechanical dyssynchrony at sea level and high altitude. Both SD-TPS [36.2 (24.5, 48.6) ms vs. 21.7 (12.9, 32.1) ms, p<0.001] and SD-TPS as percentage of R–R’ interval (4.6 ± 2.1% vs. 2.5 ± 1.8%, p<0.001) were significantly higher at high altitude than at sea level (Fig 2). Additionally, higher SD-TPSRs (%) (7.3 ± 3.5% vs. 5.9 ± 2.1%, p<0.001) was observed after high altitude exposure (Table 1). However, there were no significant differences in neither interatrial dyssynchrony (18.3 ± 14.0 ms vs. 17.1 ± 12.5 ms, p = 0.449) nor interatrial dyssynchrony as percentage (1.98 ± 1.54% vs. 2.11 ± 1.59%, p = 0.784) after high altitude exposure (S1 Fig).

**Table 1:** Right atrial dyssynchrony at sea level and high altitude.

Variables	Sea level (n = 98)	High altitude (n = 98)	P-value
SD-TPS, ms	21.7 (12.9, 32.1)	36.2 (24.5, 48.6)	**< 0.001**
SD-TPS, %	2.5 ± 1.8	4.6 ± 2.1	**< 0.001**
SD-TPSRs, ms	53.6 ± 18.5	58.8 ± 26.5	0.085
SD-TPSRs, %	5.9 ± 2.1	7.3 ± 3.5	**< 0.001**
SD-TPSRe, ms	40.1 (28.3, 54.2)	35.5 (18.6, 47.1)	0.092
SD-TPSRe, %	4.3 ± 2.2	4.3 ± 2.8	0.989
SD-TPSRa, ms	23.2 (16.8, 28.6)	20.0 (13.9, 29.2)	0.156
SD-TPSRa, %	2.4 (1.5, 3.8)	2.4 (1.7, 3.3)	0.423

Data are expressed as mean ± SD or median (25th to 75th quartile). Bold values indicate statistically significant. RA, right atrium; SD, standard deviation; TPS, time to peak strain; TPSRs, time to peak systolic strain rate; TPSRe, time to peak early diastolic strain rate; TPSRa, time to peak late diastolic strain rate.

### Characteristics of subjects according to grade of RA dyssynchrony

**Fig 3:**
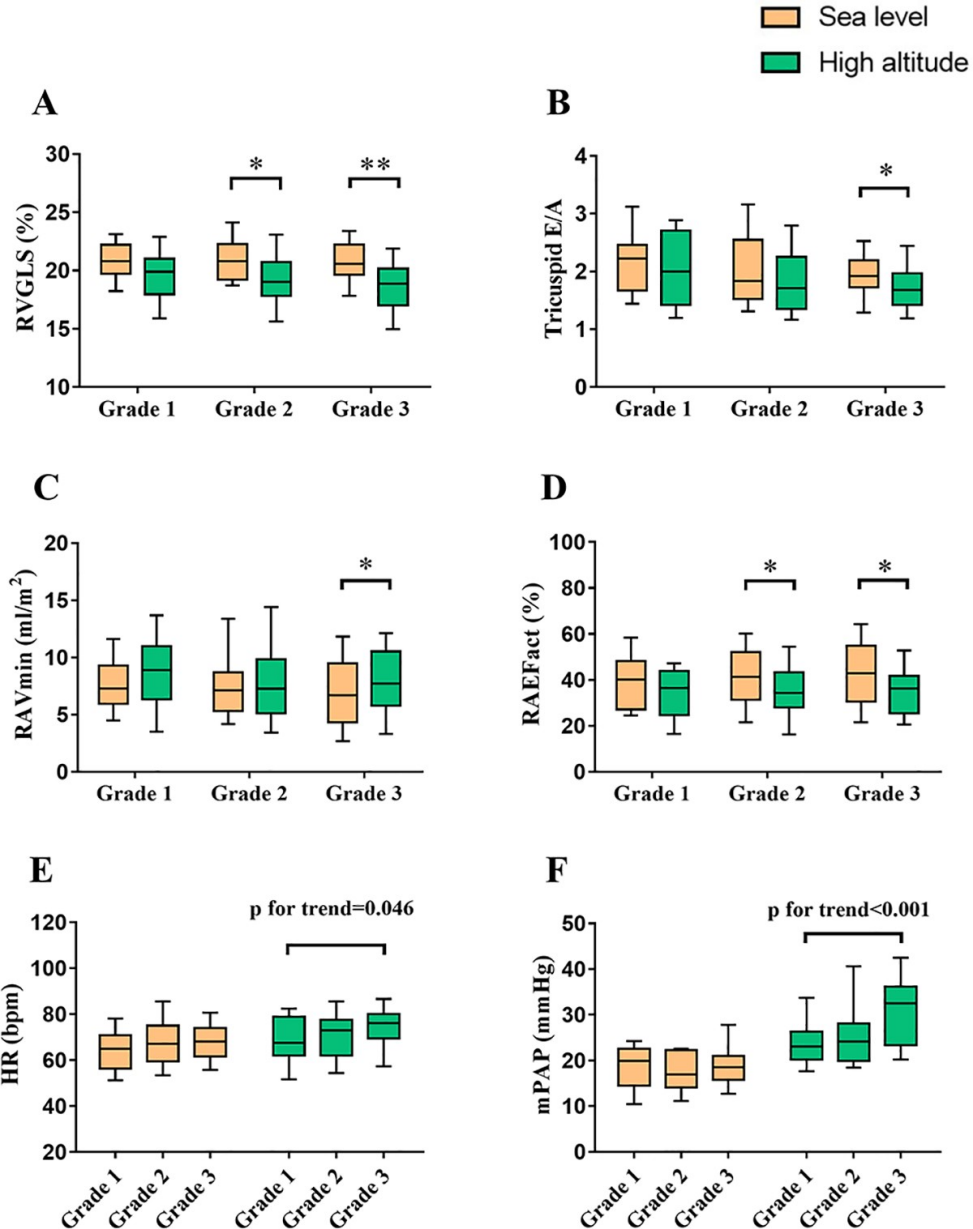
The interaction among right atrial dyssynchrony and other functions under high altitude exposure. Subjects were graded according to tertile of SD-TPS (%) value at high altitude. *p<0.05; **p<0.01. RV, right ventricle; GLS, global longitudinal strain; E/A, ratio of peak early to late diastolic annular inflow velocity; Vmin, minimal volume index; EFact, active emptying fraction; HR, heart rate; mPAP, mean pulmonary arterial pressure. Other abbreviations as in Fig 1.

Clinical and echocardiographic characteristics of subjects according to tertiles of SD-TPS (%) value at high altitude were presented in Table 2. Arterial pulse oxygen saturation significantly decreased, and blood pressure increased after high altitude exposure in all groups. Nevertheless, HR and left ventricular ejection fraction increased in Grade 3, but not in other groups. For RV parameters, fractional area change and tricuspid annular motion significantly decreased in all groups. RVGLS decreased in Grade 2 and Grade 3. In addition, tricuspid E/A decreased in Grade 3, but not in other groups. In test for trend among groups, decreasing trends of body mass index and tricuspid E/A was observed with the increase of RA SD-TPS (%) (p for trend = 0.037 and p for trend = 0.042, respectively). However, rising trends of HR and mPAP was observed with the increase of RA SD-TPS (%) (p for trend = 0.046 and p for trend<0.001, respectively) (Fig 3E and 3F). For RA parameters, EFtot and strain significantly decreased in all groups. RAEFact decreased in Grade 2 and Grade 3, but not in Grade 1. Moreover, RAVmin increased in Grade 3, but not in other groups. However, no significant difference of RAVmax and RAVpre was observed in all groups.

**Table 2:** Clinical and echocardiographic characteristics of subjects according to grade of right atrial dyssynchrony at high altitude.

Variables	Grade 1 (n = 32)			Grade 2 (n = 33)			Grade 3 (n = 33)		
	Sea level	High altitude	P-value	Sea level	High altitude	P-value	Sea level	High altitude	P-value
**Clinical parameters**									
Age, yrs	21.0 (19.0, 23.0)	—		20.5 (19.0, 22.0)	—		20.0 (19.0, 20.8)	—	
BMI, kg/m^2^	21.5 ± 1.4*	—		21.1 ± 1.9	—		20.6 ± 1.8	—	
SpO_2_, %	98.0 (96.0, 98.0)	89.0 (88.0, 90.0)	**<0.001**	98.0 (97.0, 98.0)	90.0 (88.0, 91.0)	**<0.001**	98.0 (97.0, 98.0)	89.0 (87.0, 91.0)	**<0.001**
SBP, mmHg	113.6 ± 7.4	119.8 ± 11.6	**0.002**	111.9 ± 11.3	122.0 ± 10.1	**0.002**	112.5 ± 10.7	117.7 ± 12.0	**0.034**
DBP, mmHg	67.2 ± 6.7	77.7 ± 10.0	**<0.001**	67.9 ± 9.6	80.1 ± 9.5	**< 0.001**	66.8 ± 8.1	78.0 ± 10.3	**<0.001**
HR, bpm	64.3 ± 11.1	69.0 ± 11.9*	0.060	67.8 ± 11.2	71.1 ± 10.8	0.099	68.2 ± 10.1	74.6 ± 10.7	**0.007**
**Echocardiographic parameters**									
LVEF, %	62.0 (56.1, 65.0)	63.9 (57.6, 67.9)	0.347	61.5 (50.8, 68.6)	65.0 (60.4, 69.9)	0.120	63.7 (54.4, 68.6)	66.8 (60.3, 71.9)	**0.031**
Mitral E/A	1.98 (1.52, 2.30)	1.61 (1.35, 1.82)	**0.001**	1.79 (1.49, 2.15)	1.55 (1.35, 1.90)	0.066	1.74 (1.46, 2.16)	1.53 (1.33, 1.85)	**0.010**
RV FAC,%	45.2 (43.1, 48.5)	39.6 (37.2, 43.6)	**<0.001**	42.9 (41.7, 46.3)	42.0 (41.7, 46.3)	**0.021**	46.2 (42.7, 48.2)	42.2 (37.9, 45.1)	**<0.001**
Tricuspid TAM, mm	18.4 (16.7, 19.5)	15.9 (14.6, 16.5)	**<0.001**	17.7 (16.8, 19.6)	16.2 (15.3, 17.5)	**<0.001**	18.1 (17.2, 18.9)	16.2 (14.5, 17.0)	**<0.001**
RVGLS, %	20.8 ± 1.7	19.5 ± 3.0	0.087	21.0 ± 2.0	19.2 ± 2.5	**0.018**	20.8 ± 2.1	18.5 ± 2.6	**< 0.001**
Tricuspid E/A	2.22 (1.67, 2.46)	2.00 (1.42, 2.71)*	0.335	1.83 (1.52, 2.55)	1.71 (1.35, 2.25)	0.241	1.89 (1.67, 2.15)	1.68 (1.42, 1.97)	**0.035**
TRV, m/s	2.10 (1.90, 2.37)	2.34 (2.18, 2.52)	**0.005**	2.08 (1.87, 2.34)	2.48 (2.24, 2.76)	**<0.001**	2.18 (2.02, 2.34)	2.43 (2.31, 2.97)	**0.001**
sPAP, mmHg	27.7 (24.5, 32.5)	32.0 (28.9, 35.3)	**0.030**	27.2 (24.0, 31.9)	34.5 (29.9, 40.4)	**< 0.001**	29.1 (26.4, 31.9)	33.7 (31.3, 45.2)	**0.016**
PAAT, ms	128.5 (122.5, 143.4)	112.0 (102.6, 123.7)	**<0.001**	130.7 (115.4, 144.2)	106.1 (99.9, 123.0)	**< 0.001**	134.2 (119.9, 140.6)	92.64 (86.8, 111.3)	**< 0.001**
mPAP, mmHg	19.9 (14.5, 22.6)	23.0 (20.1, 26.3)*	**0.001**	16.9 (14.1, 22.3)	24.2 (19.9, 28.1)	**0.001**	18.6 (15.7, 21.0)	32.6 (23.4, 36.2)	**< 0.001**
**RA parameters**									
RAVmax, ml/m^2^	19.0 (15.3, 21.4)	17.0 (13.7, 22.9)	0.639	19.2 (14.7, 22.5)	16.6 (13.2, 20.3)	0.062	17.2 (14.6, 21.1)	16.5 (14.5, 18.8)	0.626
RAVpre-A, ml/m^2^	13.4 ± 4.6	13.37 ± 5.6	0.966	13.4 ± 4.3	11.9 ± 4.6	0.193	12.3 ± 5.0	12.6 ± 4.9	0.741
RAVmin, ml/m^2^	8.0 (5.9, 9.3)	8.7 (6.3, 11.0)	0.351	7.1 (5.3, 8.7)	7.3 (5.1, 9.8)	0.779	6.7 (4.3, 9.5)	7.7 (5.8, 10.5)	**0.025**
RAEFtot, %	58.0 ± 9.9	53.4 ± 11.5	**0.037**	60.6 ± 11.1	54.3 ± 12.3	**0.021**	59.8 ± 13.7	53.7 ± 13.8	**0.018**
RAEFpass, %	30.0 ± 11.9	28.1 ± 11.7	0.431	30.1 ± 13.7	30.0 ± 13.7	0.721	30.4 ± 14.0	28.2 ± 14.8	0.511
RAEFact, %	40.2 (27.2, 48.2)	36.4 (24.7, 43.8)	0.276	41.4 (31.4, 52.0)	34.3 (28.0, 43.2)	**0.036**	42.9 (30.6, 54.9)	36.2 (25.5, 41.8)	**0.033**
RAS, %	43.2 ± 11.0	35.5 ± 11.4	**0.010**	44.4 ± 10.9	35.5 ± 11.4	**<0.001**	44.0 ± 9.7	35.6 ± 9.2	**<0.001**

Data are expressed as mean ± standard deviation or median (25th to 75th quartile). Bold values indicate statistically significant. *p for trend <0.05. RA, right atrium; BMI, body mass index; HR, heart rate; SpO_2_, arterial pulse oxygen saturation; SBP, systolic blood pressure; DBP, diastolic blood pressure; LVEF, left ventricle ejection fraction; E/A, ratio of peak early to late diastolic annular inflow velocity; RV, right ventricle; FAC, fractional area change; TAM, tricuspid annular motion; GLS, global longitudinal strain; PAAT, pulmonary artery systolic wave acceleration time; mPAP, mean pulmonary arterial pressure; TRV, tricuspid regurgitation velocity; sPAP, systolic pulmonary arterial pressure; Vmax, maximal volume index; Vpre-A, pre-systolic volume index; Vmin, minimal volume index; EFtot, total emptying fraction; EFpass, passive emptying fraction; EFact, active emptying fraction; RAS, right atrial strain during the reservoir phase.

### Univariable and multivariable analyses

**Fig 4:**
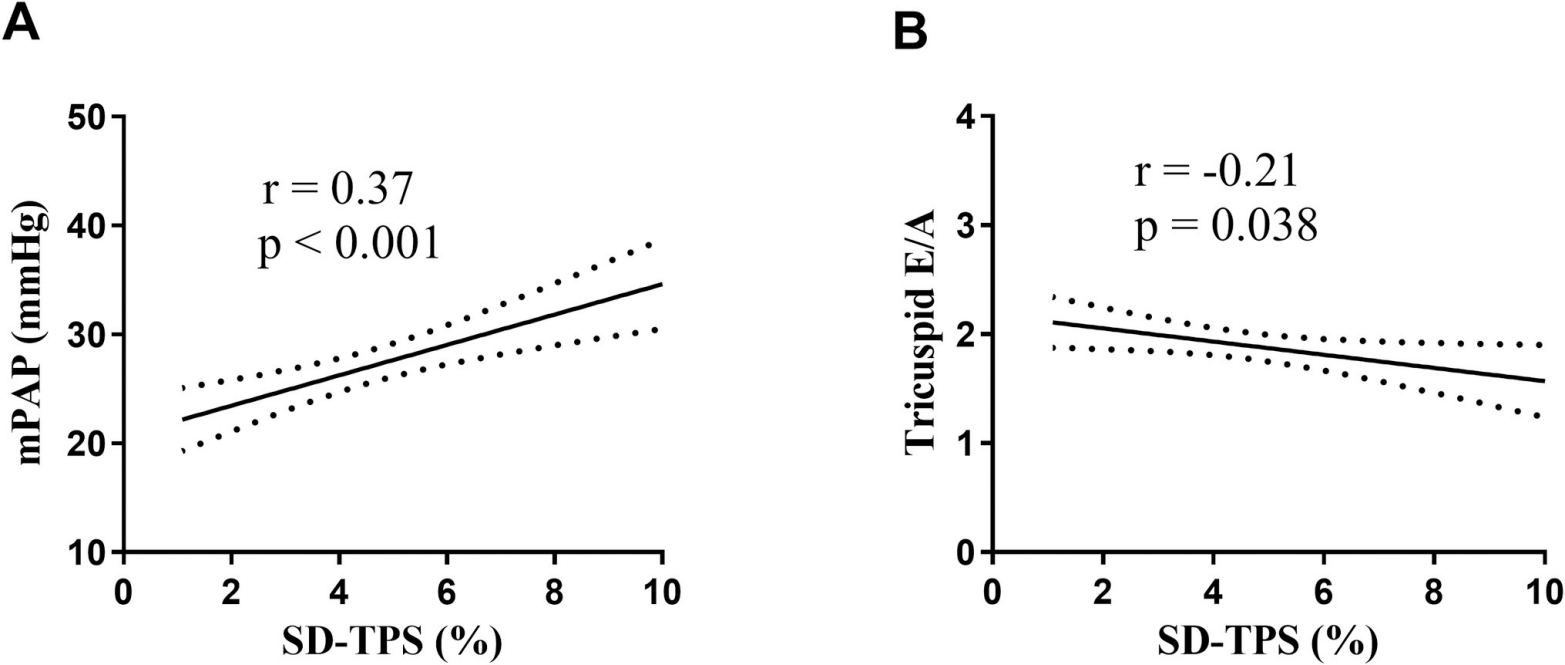
Regressions of right atrial SD-TPS (%) with mean pulmonary arterial pressure and tricuspid E/A. Abbreviations as in Figs 1 and 3.

In univariable analysis, body mass index, tricuspid E/A and mPAP at high altitude were significantly associated with SD-TPS (%). Variables with p<0.1 were entered to the stepwise multivariable linear regression. After multivariable adjustment for body mass index, HR and RVGLS at high altitude, tricuspid E/A and mPAP at high altitude were independently associated with SD-TPS (%) (β = -0.73 and β = 0.12, respectively) (Table 3). Fig 4 illustrates the regressions of RA SD-TPS (%) with mPAP (r = 0.37, p<0.001) and tricuspid E/A (r = -0.21, p = 0.038).

**Table 3:** Univariate and multivariate linear regression analyses of clinical and echocardiographic parameters for SD-TPS (%) at high altitude.

Variables	Univariate		Stepwise Multivariate	
	β (95%CI)	P-value	β (95%CI)	P-value
Age, yrs	-0.09 (-0.25, 0.08)	0.293	Not selected	
BMI, kg/m^2^	-0.32 (-0.58, -0.06)	**0.017**	-	
SpO_2_, %	-0.01 (-0.18, 0.16)	0.939	Not selected	
SBP, mmHg	-0.03 (-0.07, 0.02)	0.218	Not selected	
DBP, mmHg	0.00 (-0.05, 0.05)	0.926	Not selected	
HR, bpm	0.04 (-0.00, 0.08)	0.081	-	
LVEF, %	0.03 (-0.02, 0.09)	0.250	Not selected	
Mitral E/A	-0.59 (-1.60, 0.42)	0.248	Not selected	
RV FAC, %	0.06 (-0.07, 0.18)	0.376	Not selected	
Tricuspid TAM, mm	-0.12 (-0.36, 0.13)	0.350	Not selected	
RVGLS, %	-0.17 (-0.36, 0.01)	0.065	-	
Tricuspid E/A	-0.73 (-1.42, -0.04)	**0.038**	-0.73 (-1.37, -0.09)	**0.025**
mPAP, mmHg	0.12 (0.05, 0.16)	**<0.001**	0.12 (0.07, 0.17)	**<0.001**
sPAP, mmHg	0.05 (-0.02, 0.12)	0.143	Not selected	
RAVmax, ml/m^2^	0.00 (-0.08, 0.09)	0.898	Not selected	
RAVpre-A, ml/m^2^	0.01 (-0.08, 0.11)	0.780	Not selected	
RAVmin, ml/m^2^	0.00 (-0.13, 0.12)	0.954	Not selected	
RAEFtot, %	0.00 (-0.03, 0.04)	0.937	Not selected	
RAEFpass, %	-0.01 (-0.04, 0.02)	0.497	Not selected	
RAEFact, %	0.01 (-0.02, 0.05)	0.469	Not selected	
RAS, %	-0.01 (-0.05, 0.03)	0.632	Not selected	

Data are expressed as median (25th to 75th quartile). Bold values indicate statistically significant. Abbreviations as in Table 2.

#### Association between acute mountain sickness and atrial dyssynchrony

The incidence of acute mountain sickness in subjects according to tertiles of RA SD-TPS (%) and interatrial dyssynchrony were presented in S1 and S2 Tables, respectively. There were no significant differences in Lake Louise score and the incidences of acute mountain sickness and its related symptoms (headache, gastrointestinal symptoms, fatigue and dizziness) among all groups according to RA SD-TPS (%) or interatrial dyssynchrony Grade.

### Reproducibility

The intra-class correlation coefficients of indexes of RA function and dyssynchrony for the intra- and inter-observer variations are presented in Table 4. All measurements showed excellent or good reproducibility.

**Table 4:** Intra-class correlation coefficient analysis of intra- and inter-observer variations for right atrial function and dyssynchrony parameters.

Variables	Intra-observer variation			Inter-observer variation		
	ICC	95%CI	P-value	ICC	95%CI	P-value
RA maximal volume, ml	0.90	0.76–0.96	<0.001	0.90	0.75–0.96	<0.001
RA pre-systolic volume, ml	0.80	0.51–0.92	<0.001	0.82	0.55–0.93	<0.001
RA minimal volume, ml	0.82	0.56–0.93	<0.001	0.83	0.58–0.93	<0.001
RAS, %	0.89	0.71–0.96	<0.001	0.80	0.51–0.92	<0.001
SD-TPS, ms	0.85	0.62–0.94	<0.001	0.77	0.42–0.91	0.001
SD-TPSRs, ms	0.81	0.54–0.93	<0.001	0.80	0.51–0.92	<0.001
SD-TPSRe, ms	0.89	0.72–0.96	<0.001	0.86	0.64–0.94	<0.001
SD-TPSRa, ms	0.82	0.54–0.93	<0.001	0.82	0.52–0.93	<0.001

Abbreviations as in Tables 1 and 2.

## Discussion

In this retrospective study, we utilized a new method to explore the effect of high altitude exposure on RA mechanical synchrony by 2D speckle-tracking echocardiography. We found that high altitude exposure led to the increase of RA SD-TPS (%), which was independently associated with mPAP. Additionally, subjects with higher RA SD-TPS (%) at high altitude showed lower RVGLS, tricuspid E/A and RAEFact. For the first time, our findings indicated that high altitude exposure induced RA dyssynchrony, which was linked with decreased RA contractile function and RV performance.

In this study, 2D volumetric measurement and strain analysis were both used to assess RA function, however speckle-tracking echocardiography is less load-dependent than volume analysis and can represent regional myocardial function, especially the regional heterogeneity of myocardial motion, which provides a feasible tool to assess RA dyssynchrony at high altitude [16,21,34,35]. RA dyssynchrony can be analyzed by determining standard deviation of the time to the peak strain and peak strain rate during RA reservoir, conduit and contractile phases [7,20]. SD-TPS (%) was commonly used and has been recognized as a valuable index among above-mentioned measurements of RA dyssynchrony [7,9,36–38]. Likewise, in this study, significant increase was observed in SD-TPS (%) after high altitude exposure, while no changes in other indexes as percentage.

Previous studies have reported the increase of RA dyssynchrony and its predictive value in atrial fibrillation and heart failure patients [20,39]. Intriguingly, high altitude exposure-induced RA dyssynchrony was observed in this study. Pezzuto, Forton [40] reported that RV dyssynchrony increased after exposed to monitored 4500 m, in consequence of hypoxia. Hypoxia may be the initial determinant on RA dyssynchrony following high altitude exposure. Previous studies demonstrated that increased pressure, regional wall stress heterogeneity and delays in electrical depolarization could account for myocardial dyssynchrony [11,40,41]. Our results consistently clarified that hypoxia-induced borderline pulmonary hypertension caused pressure overload to RA, and led to RA dyssynchrony. However, high altitude exposure induced pronounced RA intra-atrial dyssynchrony, but didn’t prolong interatrial dyssynchrony. Additionally, we observed that higher HR was related to higher RA dyssynchrony at high altitude, which implied that sympathetic activation may involve in high altitude exposure-induced RA dyssynchrony.

RA function has three phases, serving as a reservoir during systole, as a conduit during early diastole, and as a booster pump during late diastole. In the present study, subjects with higher RA dyssynchrony showed decreased RA contractile function (assessed by RAEFact) after high altitude exposure. However, no differences of RA reservoir and conduit function were found in subjects with different magnitude of RA dyssynchrony. Indeed, atrial reservoir and conduit function were considered to be regulated by RA and RV relaxation and compliance [16]. Badagliacca, Poscia [11] indicated that RV dyssynchrony was related to RV systolic dysfunction in patients with pulmonary arterial hypertension, due to a maladaptive switch of myosin heavy chain. Similarly, previous immunofluorescence studies have observed a transition from α- to β-myosin heavy chain with lower adenosine triphosphatase activity in pressure-overloaded atrium [42,43]. Likewise, the mechanism may involve in the high altitude-induced RA dyssynchrony and subsequently decreased RA contractile function, which need to be clarified at protein expression level.

It must be acknowledged that interplay exists among atrial function and ventricular performance throughout the cardiac cycle [44]. RA effective contraction participates in the final component of RV diastole and contributes approximately 15%-30% to RV stroke volume [45]. Accordingly, it is easy to understand that high altitude exposure causes pronounced RA dyssynchrony and atrial inhomogeneous contraction, which may ultimately alter RV filling pattern (assessed by tricuspid E/A). Moreover, RVGLS strain significantly deceased in subjects with higher RA dyssynchrony. It is not unexpected that decreased RV longitudinal systolic function causes excessive residual blood and increased pressure during diastole, which may conduct to RA and ultimately aggravate RA dyssynchrony. Our results suggested that RA dyssynchrony may interact with RV systolic as well as diastolic performance.

Our study may provide a novel insight into cardiac response to high altitude, but the clinical implication remains to be clarified in the further study. Hypoxia, alkalosis and pulmonary hypertension may predispose to atrial arrhythmia at high altitude, but evidence is limited due to the difficulty in the field study at high altitude [1,4]. Increasing studies have demonstrated that mechanical dyssynchrony was directly linked with arrhythmia [9,37]. In the present study, we observed that high altitude exposure led to pronounced RA mechanical dyssynchrony, which probably provide a possible atrial mechanical mechanism underlying high altitude-induced arrhythmia. Additionally, it is well established that high altitude exposure is accompanied by decreased exercise capacity [2]. Indeed, Liu, Wang [46] that RA dysfunction was a risk factor for worse exercise capacity in patients with pulmonary hypertension. Accordingly, our findings might imply a relationship between increased RA dyssynchrony and decreased exercise capacity at high altitude.

However, there are still several limitations in the present study. First, the data is collected from healthy young men. Thus, larger population with wider scale of age, different genders and cardiovascular diseases should be included in further study. Second, as limited studies have explored RA dyssynchrony in healthy subjects or patients using 2D speckle-tracking echocardiography, no reference value can be used to make a comparison. Third, 3D speckle-tracking was not included in this study since it is not widely used and time-consuming. Although 2D speckle-tracking echocardiography to assess atrial function overcomes the volume- and angle-dependency of traditional echocardiography, 3D speckle-tracking is still needed in the further study to clarify the results. Fourth, invasive cardiac catheterization and in vitro experiment should be added to validate the results and further illustrate the mechanism. Finally, longer follow-up is needed to clarify the clinical relevance of our results.

## Conclusion

This study for the first time investigated the effect of high altitude exposure on RA mechanical dyssynchrony in healthy young men. The result showed that high altitude exposure causes RA dyssynchrony, which may be secondary to increased pulmonary arterial pressure. Additionally, subjects with higher RA dyssynchrony presented worse RA contractile function and RV performance. Our findings perhaps can provide a potential atrial substrate for the high altitude-induced arrhythmia. However, the pathophysiology and the clinical relevance remain to be explored in further study.

## Supporting information

S1 Checklist STROBE_checklist_v4_combined_PlosMedicine. (DOCX)

S1 Dataset Raw data. (XLSX)

S1 Fig Comparison of interatrial dyssynchrony between at sea level and high altitude. (TIF)

S1 Table The incidence of acute mountain sickness in subjects according to grade of right atrial dyssynchrony at high altitude. (DOCX)

S2 Table The incidence of acute mountain sickness in subjects according to grade of interatrial dyssynchrony at high altitude. (DOCX)
