# Supplementary figures and images for: Assessment of right atrial dyssynchrony by 2D speckle-tracking in healthy young men following high altitude exposure at 4100 m
Source: PLoS One. 2021 Feb 18;16(2):e0247107. doi: 10.1371/journal.pone.0247107 (PMC7891700; doi:10.1371/journal.pone.0247107)

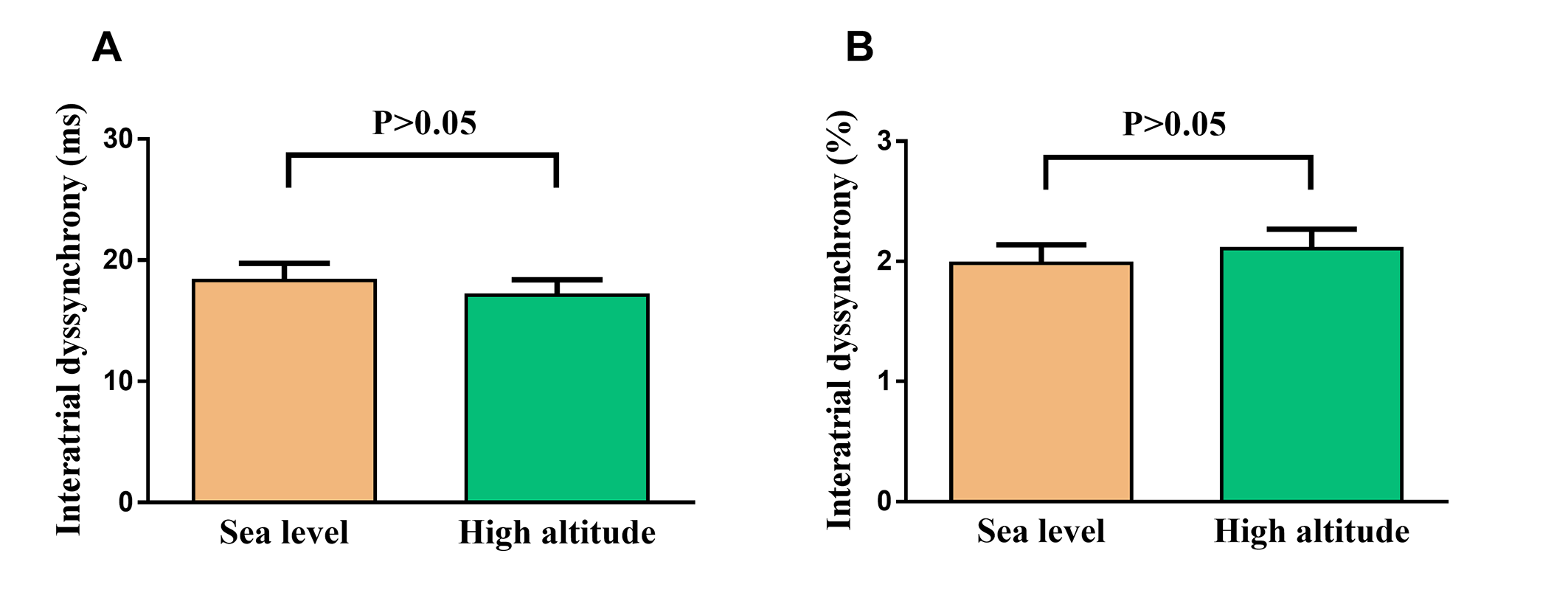

Supplement: S1 Fig — (TIF) [file pone.0247107.s003.tif]
