# Supplementary material for: Assessment of right atrial dyssynchrony by 2D speckle-tracking in healthy young men following high altitude exposure at 4100 m
Source: PLoS One. 2021 Feb 18;16(2):e0247107. doi: 10.1371/journal.pone.0247107 (PMC7891700; doi:10.1371/journal.pone.0247107)
Supplement: S2 Table — (DOCX) [file pone.0247107.s005.docx]

**S2 Table. The incidence of acute mountain sickness in subjects according to grade of interatrial dyssynchrony at high altitude.**

| **[Symptom](C:/Users/xianghedong/AppData/Local/youdao/dict/Application/8.9.3.0/resultui/html/index.html#/javascript:;)s** | **Grade1** (n=32) | **Grade2** (n=33) | **Grade3** (n=33) | **P-value** |
| --- | --- | --- | --- | --- |
| Acute mountain sickness, n (%) | 10 (31.2%) | 13 (39.3%) | 10 (30.3%) | 0.692 |
| Lake Louise score | 2.00 (1.00, 3.00) | 2.00 (0.00, 3.00) | 1.00 (0.00, 3.00) | 0.830 |
| [Headache](C:/Users/xianghedong/AppData/Local/youdao/dict/Application/8.9.3.0/resultui/html/index.html#/javascript:;), n (%) | 15 (46.8%) | 19 (57.6%) | 15 (45.4%) | 0.561 |
| Gastrointestinal symptoms, n (%) | 3 (9.3%) | 2 (6.0%) | 4 (12.1%) | 0.717 |
| Fatigue, n (%) | 18 (56.2%) | 17 (73.9%) | 16 (48.5%) | 0.820 |
| Dizziness, n (%) | 19 (59.3%) | 16 (48.5%) | 17 (12.1%) | 0.663 |

Data are expressed as median (25th to 75th quartile) for continuous variables, and the number with proportions for categorical variables.
